# Effectiveness of Written Dietary Advice for Improving Blood Lipids in Primary Care Adults—A Pragmatic Randomized Controlled Trial (MYDICLIN)

**DOI:** 10.3390/nu14051022

**Published:** 2022-02-28

**Authors:** Andreas Rydell, Mikael Hellsten, Martin Lindow, David Iggman

**Affiliations:** 1 Norslund-Svärdsjö Academic Primary Health Care Center, Region Dalarna, Svärdsjö Vårdcentral, Björkvägen 2, S-790 23 Svärdsjö, Sweden; andreas.rydell@regiondalarna.se (A.R.); mikael.hellsten@regiondalarna.se (M.H.); 2 Department of Neurobiology, Care Sciences and Society, Division of Family Medicine and Primary Care, Karolinska Institutet, Alfred Nobels Allé 23, S-141 83 Huddinge, Sweden; 3 Center for Clinical Research Dalarna—Uppsala University, Nissers Väg 3, S-791 82 Falun, Sweden; 4 School of Medical Sciences, Örebro University, Campus USÖ, S-701 82 Örebro, Sweden; martin.lindow@regionorebrolan.se; 5 Clinical Nutrition and Metabolism, Department of Public Health and Caring Sciences, Uppsala University, Husargatan 3, S-752 37 Uppsala, Sweden

**Keywords:** LDL cholesterol, triglycerides, lipids, primary care, foods, dietary advice

## Abstract

Lifestyle management is the first line of treatment for moderately elevated blood lipids in healthy individuals. We investigated the effectiveness of providing food-based written advice for lowering low-density lipoprotein (LDL) cholesterol (intervention) or triglycerides (control) in a pragmatic randomized controlled trial with two parallel arms from 2018–2019 at a rural primary health care center. We sent feedback letters after 3 weeks and 6 months. Out of the 113 adult primary care patients randomized, 112 completed the study. There were no differences between the intervention and control groups for changes in LDL cholesterol after 3 weeks (mean ± standard deviation −0.21 ± 0.38 vs. −0.11 ± 0.34 mmol/L, *p* = 0.45) or 6 months (−0.05 ± 0.47 vs. 0.02 ± 0.41 mmol/L, *p* = 0.70) (primary outcome). Following the advice to consume plant sterols and turmeric was associated with a reduction in LDL cholesterol after 3 weeks. Following the advice to consume less carbohydrates was associated with reduced triglycerides. In the intervention arm, 14 individuals (25%) reduced their LDL cholesterol by ≥10% after three weeks. Their reduction was attenuated but maintained after six months (−7.1 ± 9.2% or −0.31 ± 0.38 mmol/L, *p* = 0.01 compared with baseline). They differed only in higher adherence to the advice regarding turmeric. In conclusion, this undemanding intervention had little effect on blood lipids for most individuals.

## 1. Introduction

Dyslipidemia causally contributes to atherosclerotic cardiovascular disease, which is a leading cause of disability and premature death. For individuals with moderately elevated blood lipids but without previous cardiovascular disease, pharmacological treatment is not always indicated. They may instead primarily benefit from lifestyle modification. As health care resources are limited, practical and effective methods for providing dietary guidance are warranted.

In the guidelines for treating dyslipidemia, much focus is on improving LDL cholesterol or related surrogate risk measures, e.g., non-HDL cholesterol or apolipoprotein B [1,2]. LDL cholesterol has been extensively studied and has widespread clinical utility. It is considered to causally affect the risk of future cardiovascular disease with a cumulative effect over time, i.e., the higher the levels and the longer the exposure, the greater the risk [1,3]. Elevated triglycerides may causally contribute to atherosclerosis, but the evidence is more ambiguous than for LDL cholesterol [1,4]. Importantly, dietary changes that improve LDL cholesterol [5] do not necessarily affect triglycerides, and vice versa [1]. For LDL cholesterol, combinations of dietary changes have produced pronounced effects (up to 30% reductions) in strictly controlled metabolic ward studies with high compliance. One such example is the Portfolio Diet, which emphasizes the increased intake of foods with added plant sterols, viscous fibers, soy foods, and nuts [6]. If information delivered by health care personnel could facilitate patients to perform several effective dietary changes simultaneously, this should be enough to produce measurable and clinically meaningful effects.

Previous attempts to improve blood lipids in primary care settings have shown only modest long-term effects on LDL cholesterol [7,8]. Predictably, interventions of a higher intensity produce larger effects [9]. Nevertheless, some low-intensity interventions have managed to demonstrate clear effects, using mostly printed material [10]. Advice from dieticians seems to have a larger impact than that from physicians, but it is still not clearly superior to written instructions [11]. A systematic review from 2018 concluded that various methods for remotely delivering intervention using self-monitoring or tailored feedback produced small but significant effects on eating behavior [12].

In our clinical experience, effects on blood lipids in patients performing dietary changes have varied substantially, which may depend upon baseline blood lipid levels, habitual diets, compliance, gut microbiota, and genetic factors [1]. We aimed to provide detailed and at least partly novel information to the participants, allowing them to self-select among the pieces of advice and then receive feedback on their performed changes in the spirit of person-centered care and personalized medicine [13], providing an opportunity to perform behavioral changes through education, persuasion, and incentivization [14]. Our hope was that in successful cases (≥10% reductions) the positive feedback after three weeks would help maintain the new dietary habits. The research hypothesis was that the provided written advice would cause greater improvements in LDL cholesterol than the control advice. We thus aimed to investigate whether a brief, minimalistic intervention, which was undemanding for the caregiver but instead relied mostly on patient involvement, could be a feasible and effective method to improve blood lipids in both the short and medium term in an outpatient primary care setting.

## 2. Materials and Methods

### 2.1. Study Participants

The study was conducted from May 2018 to April 2019. Volunteers were recruited through advertisements at the Svärdsjö Primary Health Care Center and in the nearby rural area, in the local advertisement bulletin, and in local social media groups. Inclusion criteria were: patients listed at the health care center who were willing to improve blood lipids and were aged 18–99 years. Exclusion criteria were: medications affecting lipid metabolism (statins, ezetimibe, fibrates, PCSK9 inhibitors, neuroleptics, cortisone, amiodarone, estrogen, progesterone, testosterone, cyclosporin, tacrolimus, loop diuretics, protease inhibitors, and anticonvulsants; beta-blockers, thiazide diuretics, and SGLT2 inhibitors were accepted at stable use), malignant disease, extreme diet (vegan diet, strict low carbohydrate diet, other ongoing weight loss diet), disturbance in metabolism (e.g., untreated hypothyroidism or hyperthyroidism), dementia or inability to understand written instructions in Swedish, other participant from the same household, or current employment at the health care center.

### 2.2. Randomization, Allocation Concealment, and Blinding

The participants were randomized 1:1 at the first visit (day −3 or the preceding days) by receiving sequentially numbered, opaque envelopes containing the dietary advice unique to their study arm. For eligible individuals from the same household, participation was decided by coin toss. The envelope was opened after the visit, to retain investigator allocation concealment throughout the study. The allocation sequence (and its block sizes) was provided by an external agent (Uppsala Clinical Research Center) and was not revealed until data collection was finished. The feedback letters were selected and posted by the assistant nurse who performed blood sampling and measurements but was not otherwise involved in the research. The participants were blinded to the study hypothesis, i.e., they were unaware whether they were allocated to the intervention or control arm. However, the nature of the study did not allow for blinding of which dietary advice to follow.

### 2.3. Dietary Intervention and Investigations

The dietary advice given in the study envelopes was printed on both sides of a sheet of A4 paper. One side contained instructions and advice, including estimates of the expected effects of different foods and their potential mechanisms, Supplementary Figure S1. The other side contained a table (here annotated as “dietary advice diary”) in which self-selected adherence to dietary advice was instructed to be indicated daily (“make an X”) during the first three weeks (days 0–20) to reflect which pieces of dietary advice participants chose to begin incorporating into their diets for how many of the first 21 days. Both sides contained components of motivational interviewing (translated from Swedish: “Great that you want to improve your blood lipids! Which pieces of advice suit you? How low can you go? Why I want to improve my blood lipids (write down your three main reasons):”), as well as designated spaces to write down the test results after three weeks and six months (for LDL cholesterol in the intervention group and for triglycerides in the control group). The three pieces of advice in the control group for reducing triglycerides were directly obtained from dyslipidemia guidelines [1], whereas the 13 pieces of advice in the intervention group were derived from the tentative results of a concurrently (2018–2019) performed systematic review of the effects of foods on LDL cholesterol [5]. In short, the advice included all foods for which an effect on LDL cholesterol had been reported in previously published systematic reviews without taking the quality of evidence into account. We did not provide any foods and avoided mentioning specific brand names. We performed no assessments of actual dietary intakes (e.g., food records) before or during the study in order to maximize the utility of the study by not introducing observational bias, i.e., to avoid affecting (restricting) the dietary intakes of the participants, beyond the actual intervention [15]. Instead, we chose a dietary advice diary simple enough to be applicable in primary care clinical practice while still providing documentation about adherence, as well as a reminder to participants when evaluating their feedback. We assessed adherence to dietary advice during the first three weeks as the number of days (0–21) with reported altered dietary habits in the dietary advice diary. We noted the number of reported reasons for wanting to improve blood lipids and categorized them as being related to cardiovascular disease, health and wellbeing, or other and personal.

The primary outcome was change in LDL cholesterol. Secondary outcomes included other lipids and risk factors, proportion of responders at 3 weeks (≥10% reduction in LDL cholesterol from baseline), the effect in these responders after 6 months, which food choices were associated with effects, the stated reasons for wanting to reduce blood lipids, and their association with effects. We drew blood samples after an overnight fast on days −3, 0 (start of intervention), 18, 21, 180, and 183. On days –3, 18, and 180, only LDL cholesterol was measured (fasting or non-fasting) in order to provide mean values from duplicate samples, which reduced the error due to day-to-day variability from approximately 7% to 5% [16]. On days 0, 21, and 183, all planned blood samples and anthropometric and blood pressure measurements were taken, Graphical abstract. We measured weight in light clothing and waist circumference between the lowest rib and the iliac crest at the end of an expiration, in accordance with local routines. We measured blood pressure in a sitting position after five minutes of rest using an Omron M6 AC. Blood samples were analyzed at the county hospital, Falu Lasarett, using standard methods (details at URL: https://www.regiondalarna.se/plus/vard/laboratoriemedicin/, accessed on 1 October 2021) on the same day or were kept cool and analyzed on the following day if drawn in the afternoon. LDL cholesterol was analyzed using a direct method (P-LDL-Kolesterol SWE05408), with the coefficient of variation of 1.5% at 3.9 mmol/L. We defined diabetes as prior diagnosis or at least two measurements of fasting plasma glucose levels ≥7.0 mmol/L or HbA1c ≥ 48 mmol/mol, and we defined impaired fasting glucose as fasting plasma glucose levels of 6.1–6.9 mmol/L.

After the first three weeks (designated as “active intervention,” Graphical abstract), and after 6 months, we sent to participants standardized letters containing lab test results and feedback depending on whether their LDL cholesterol had not improved (≤0%), had improved modestly (0.1–9.9%), or had improved clearly (≥10% reduction), Supplementary Table S1. We sent similar feedback letters to the control group, focusing on triglycerides instead.

### 2.4. Clinical Trial Registry and Ethics

The trial was registered in the Clinicaltrials.gov (accessed on 22 December 2021) database with the identifier NCT03528252. The study was approved by the Regional Ethics Committee in Uppsala, Dnr 2018/119, 2018-04-04, and conducted in accordance with the ethical standards of the Helsinki Declaration. The participants provided written informed consent and received a small monetary compensation (500 SEK, ~50 €) after completion of the study.

### 2.5. Statistical Methods

We based the power calculation on data from previous randomized intervention studies of dietary fat quality on lipoproteins [17,18], giving duplicate samples as described above. Assuming 20% loss to follow-up in the intervention group and 5% cross-over in the control group, at least *n* = 222 participants were required for alpha = 0.05 and beta = 0.10 in order to detect an effect of 0.175 ± 0.30 mmol/L (~5%) in the primary outcome variable of LDL cholesterol (after 6 months). We used the Sealed Envelope power calculator available at URL: www.sealedenvelope.com/power/continuous-superiority/, accessed on 1 October 2021. The chosen effect size was considered to be of borderline clinical relevance, equivalent to day-to-day variability (having duplicate samples), and slightly exceeding most previous low-intensity interventions. Mainly due to a lack of funding, in September 2018 when 81 individuals had been randomized but without access to study data, we performed an updated, formative power calculation. We concluded that 106 participants were required to complete the study for alpha = 0.05 and beta = 0.20 for the same effect, also allowing for 5% cross-over effect in the control group. As the loss to follow-up had been considerably lower than expected, we chose to terminate recruitment on 16 October 2018. We uploaded the statistical analysis plan to the study registry before the data collection was finished and the allocation sequence was broken. Two researchers independently transferred data from electronic medical records to IBM SPSS 26 and cross-checked data for inconsistencies in accordance with a prespecified data management plan. The results were analyzed as intention-to-treat by conservatively imputing baseline data when data were missing at follow-up. We checked variables for normality by visual inspection of histograms and QQ plots, and we performed sensitivity analyses for skewed distributions using logarithmic values. Such variables (baseline age, ALT, HbA1c, glucose, and triglycerides) are presented as medians (interquartile range, IQR) rather than means (standard deviations, SD). For between-group comparisons, we performed general linear models (or logistic regression for dichotomous variables) adjusted for factors a priori considered important (baseline values, BMI, age, and sex). The underlying assumptions of the model were assessed by examining residuals. For sensitivity analyses, we also performed unadjusted, non-parametric tests (Mann–Whitney U test). We performed comparisons with baseline values as paired *t*-tests. We analyzed the associations between the number and type of reasons given and adherence to dietary advice using the Mann–Whitney U test. Comparisons between baseline characteristics were analyzed using Fisher’s exact test for categorical variables and independent sample *t*-tests for continuous data. Linear regressions were unadjusted and included all data. We considered *p* < 0.05 to be statistically significant.

## 3. Results

**Figure 1:**
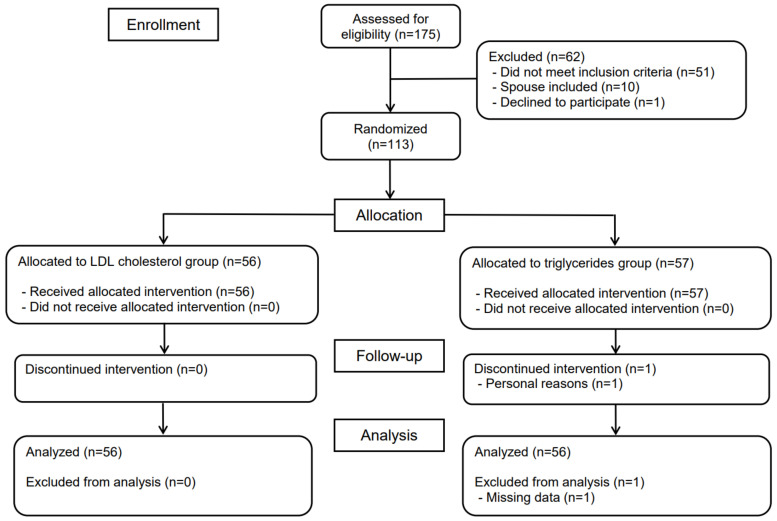
Flow diagram of the phases of the parallel randomized trial.

**Figure 2:**
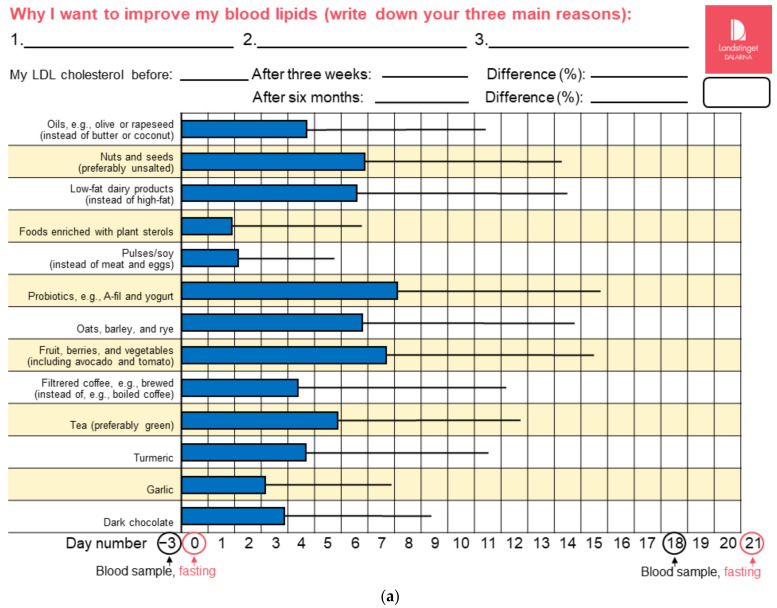

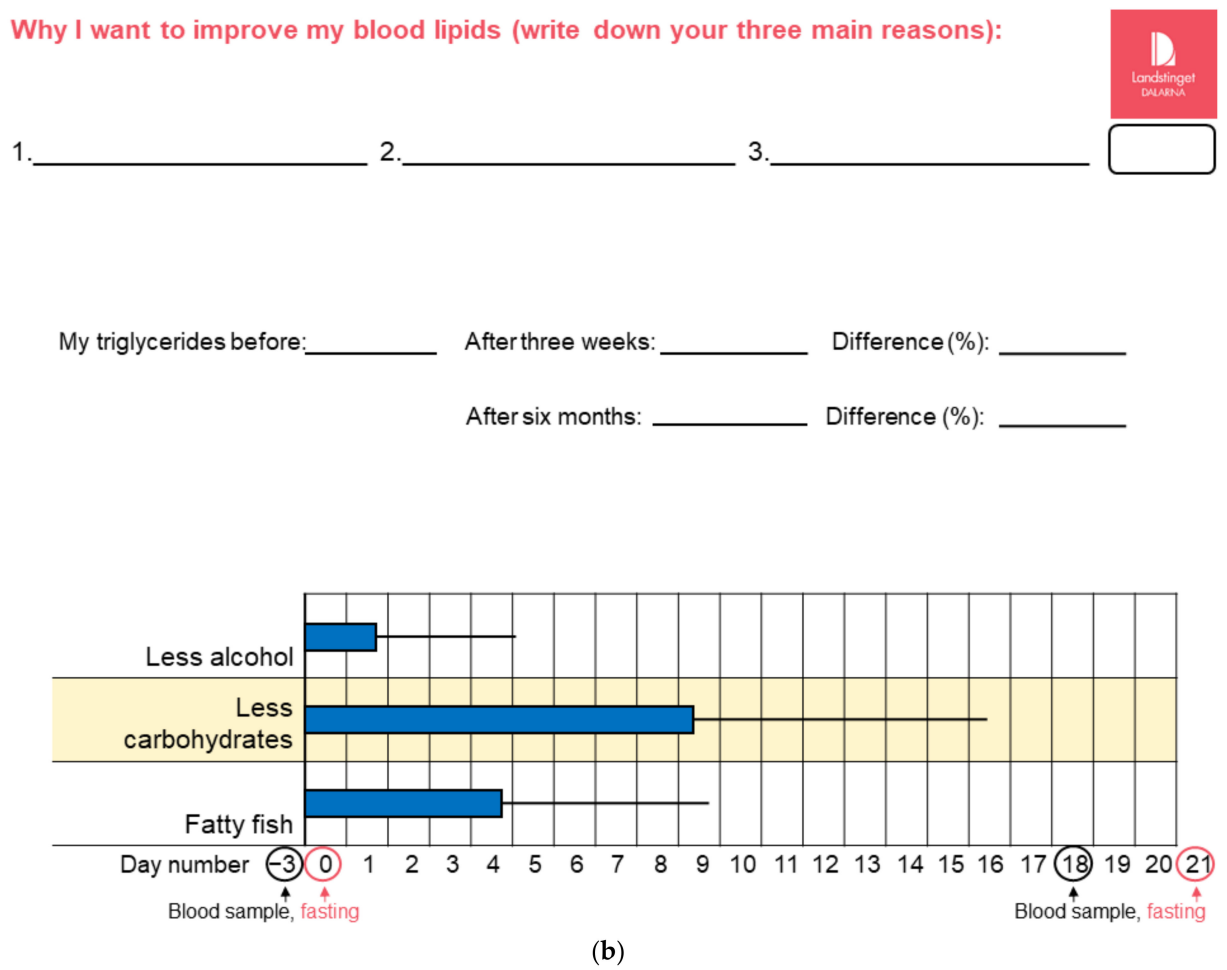
Self-reported adherence to dietary advice during active intervention (first 21 days). Adherence denotes changes compared with habitual intakes, i.e., the dietary habit was not preexisting. (**a**) Intervention group. (**b**) Control group. Boxes and whiskers represent means and SD in the number of days with reported adherence.

Baseline characteristics are presented in Table 1. The majority of the 113 participants were female (69%). They were on average 64 (range 24–88) years, overweight, and hypercholesterolemic. One of the 57 participants in the control group was lost after receiving allocation and the initial blood sample at day −3. All others (*n* = 56 in each group) completed the study, Figure 1. There were no reported adverse events and no important deviations from protocol. Self-reported adherence to dietary advice (changes from preexisting habits) during the first 21 days is presented in Figure 2. Participants in the intervention and control groups reported overall adherence to advice for (mean ± SD) 67 ± 46 and 16 ± 11 days, respectively, representing 25% maximal adherence for both groups. This would approximately correspond to following 3 out of 13 possible pieces of advice for every day in the intervention group. However, most individuals instead reported adherence to numerous pieces of advice during fewer days. Advice to consume foods enriched with plant sterols and pulses or soy had the lowest adherence, whereas probiotics and the category of fruits, berries, and vegetables had the highest.

**Figure 3:**
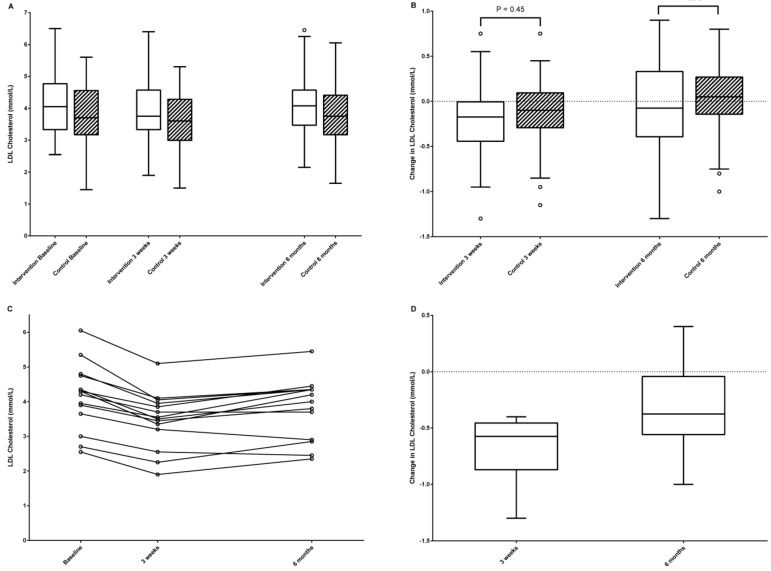
Effects on LDL cholesterol. (**A**) LDL cholesterol levels by study group. (**B**) Changes in LDL cholesterol by study group. *p*-values are from general linear models and are adjusted for baseline values, BMI, age, and sex. (**C**) LDL cholesterol levels in intervention group responders. (**D**) Changes in LDL cholesterol in intervention group responders. In (**A**,**B**,**D**), outliers >1.5 interquartile range from the nearest quartile are given as circles (if any). *n* = 14 individuals in the intervention arm with ≥10% reduction after 3 weeks.

Comparisons between groups after three weeks and six months are presented in Table 2 and Figure 3. There was no difference between groups for changes in LDL cholesterol, other lipids, glucose, or blood pressure. Fourteen individuals (25%) in the intervention group were classified as responders; however, this was not significantly different (*p* = 0.47) compared with the 10 individuals (18%) who correspondingly showed a decrease in LDL cholesterol ≥10% in the control group after 3 weeks. After 6 months, 10 individuals (18%) in the intervention group and 11 (19%) in the control group had decreased in LDL cholesterol ≥10% from baseline (*p* = 0.68). The decrease in LDL cholesterol among the 14 responders in the intervention group after three weeks (−16.2 ± 5.1%) was attenuated but maintained (−7.1 ± 9.2%, *p* = 0.01) after a six month follow-up. In exploratory post hoc analyses for this group, similar decreases (*p* ≤ 0.02) were demonstrated for total cholesterol and apolipoprotein B at 3 weeks and 6 months, in the apolipoprotein AI:B ratio at 3 weeks (*p* = 0.001), and in HDL cholesterol at 6 months (*p* = 0.01), but not for weight or waist circumference (*p* ≥ 0.08) and less consistently for systolic (−9 ± 15 mmHg after 6 months, *p* = 0.04) or diastolic blood pressure (−6 ± 9 mmHg after 3 weeks, *p* = 0.04). Adherence to increased turmeric intake was more common in responders than non-responders: a median (IQR) of 11 (0–16.5) vs. 0 (0–4) reported daily changes, *p* = 0.005. There was a non-significant tendency also for higher adherence to increased (green) tea intake in responders: 10.5 (0–14) vs. 1.5 (0–8.25), *p* = 0.07. No baseline characteristics differed between responders and non-responders (*p* ≥ 0.11). Post hoc analyses demonstrated no significant interactions (*p* > 0.31) between the covariates included in the general linear model.

Body weight and BMI were slightly reduced in the control group after three weeks, but this was no longer significant after six months or in sensitivity analyses using non-parametric tests (*p* = 0.13 for weight and *p* = 0.17 for BMI). Other results were robust to sensitivity analyses using non-parametric methods. Changes from baseline to three weeks were significant for LDL cholesterol within both the intervention (*p* < 0.001) and control (*p* = 0.02) groups, but not from baseline to six months (*p* = 0.46 and *p* = 0.71). For triglycerides, there were no significant changes from baseline (*p* > 0.37).

**Figure 4:**
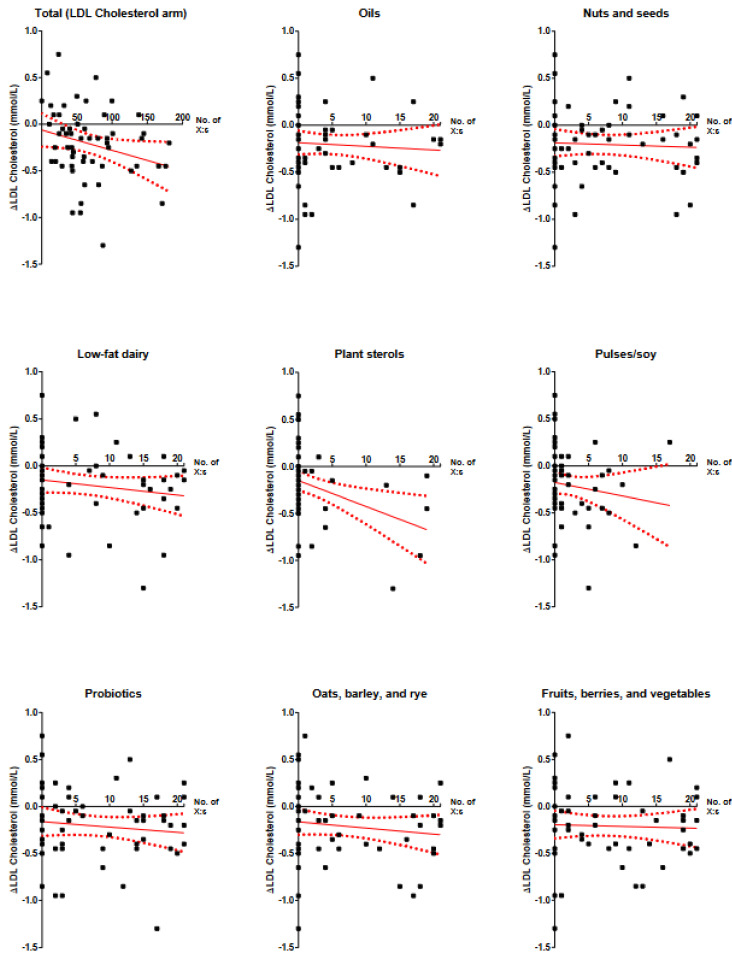

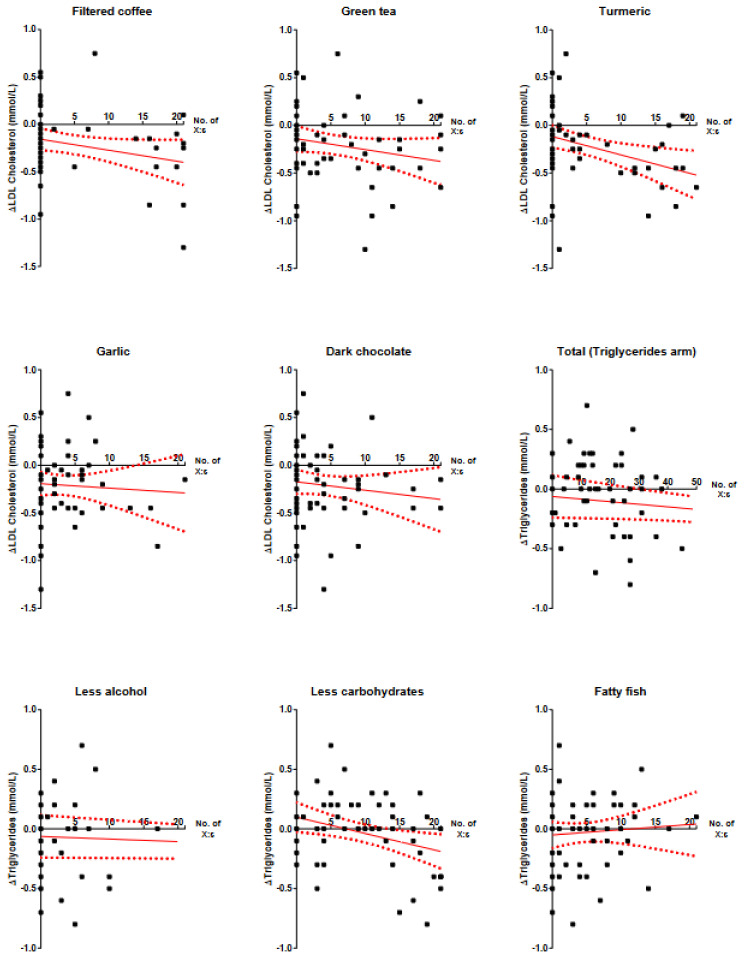
Linear regressions of self-reported adherence to dietary advice a and change in LDL cholesterol (intervention group) or triglycerides (control group) at three weeks. Dashed lines represent 95% confidence intervals for the regression lines. X denotes the number of days with indicated adherence. Adherence denotes changes compared with habitual intakes, i.e., the dietary habit was not preexisting.

Associations between self-reported adherence to dietary advice and change in LDL cholesterol (or triglycerides for the control group) are presented in Table 3 and Figure 4. For overall adherence (the total number of reported daily changes) at three weeks, the β-coefficient was −0.002 (−0.004, 0.000), *p* = 0.051. Among individual pieces of advice, those concerning plant sterols (−0.027 (−0.047, −0.007), *p* = 0.009) and turmeric (−0.019 (−0.034, −0.005), *p* = 0.01) were associated with decreased LDL cholesterol at three weeks, but not after six months. After six months, only low-fat dairy was associated with decreased LDL cholesterol (−0.016 (−0.032, −0.000), *p* = 0.048). For the replacement of unfiltered Scandinavian-style boiled coffee with filtered (brewed) coffee, there was a non-significant tendency towards reduced LDL cholesterol at both three weeks (−0.012 (−0.025, 0.001), *p* = 0.08) and six months (−0.015 (−0.031, 0.000), *p* = 0.06). In the control group, reported adherence to the advice to eat less carbohydrates was associated with reduced triglycerides at three weeks (−0.014 (−0.024, −0.003), *p* = 0.01) but not after six months.

In the two groups combined, 58 individuals (51%) reported three reasons for wanting to improve their blood lipids, 18 (16%) reported two reasons, 8 (7%) reported one reason and 29 (26%) reported no reason (or missing data, *n* = 1). In the intervention group, only individuals who reported three reasons (*n* = 29, 52%) had a significant decrease in LDL cholesterol after three weeks (mean, 95% confidence interval (CI): −0.28, −0.43 to −0.13 mmol/L, *p* < 0.001). Among the categories, health and wellbeing was the most common (*n* = 61, 54%), followed by cardiovascular disease (*n* = 50, 44%) and other or personal reasons (*n* = 47, 42%). Individuals who reported more than one reason had higher self-reported adherence to advice (29% of all potential daily changes, *p* = 0.02 and *p* = 0.001 for two and three reasons, respectively) than individuals who reported no reason (18%), but no such association was observed for individuals reporting only one reason (12%, *p* = 0.70).

## 4. Discussion

In this pragmatic randomized controlled trial, written dietary advice combined with standardized feedback was overall ineffective in improving LDL cholesterol. Although most individuals reported at least partly adapting to several of the provided pieces of advice, only 25% in the intervention group succeeded in improving their LDL cholesterol by at least 10%, which is still inferior to pharmacological agents. There were considerable individual differences in both the self-selection of dietary advice and in their associations with effects on blood lipids.

The effects on LDL cholesterol after 6 months were lower than in other studies performed in primary care settings (−0.16, −0.24 to −0.08 mmol/L in a meta-analysis of 17 study arms with 3–24 month durations) [7]. However, those studies compared dietary advice with no or minimal advice, i.e., intensity was generally lower in the control arms [19,20,21,22,23,24,25,26,27,28,29,30,31]. Another related study was performed simultaneously in a UK primary care setting investigating the effects of brief support (verbal and written advice) and personalized feedback on grocery shopping, with a focus on the reduction of saturated fat intake. It also failed to clearly improve blood lipids compared with control after three months [32].

Among the given pieces of dietary advice, the short-term effect of foods with added plant sterols seems promising, but the low adherence and lack of effect at six months raises some concerns about its applicability in this population. Conceivably, such advice should clearly define available food products to increase adherence. The short-term effect of turmeric intake and the non-significant tendencies towards the short- and medium-term effects of replacing unfiltered with filtered coffee indicated that these dietary changes may be attainable and effective for certain individuals. This was as also indicated by the self-reported adherence (high proportion of high numbers) for filtered coffee intake in place of unfiltered (Figure 4). A larger sample size or meta regression would be required to confirm this hypothesis. In the control group, there was some indication that consuming less carbohydrates might be more pragmatically effective for improving triglycerides than reducing alcohol intake or increasing fatty fish intake. However, we did not assess baseline intakes of carbohydrates, fish, or alcohol. Also, these participants had normal baseline triglycerides and may have been more health-conscious than the general population with lower habitual alcohol intakes. It is conceivable that motivational strategies may be ineffective in the present written format, and face-to-face discussion is required with an empathetic caregiver [33]. Also, motivational interviewing in itself may not be superior to standard care for dyslipidemia [34].

It was somewhat surprising that several well-established pieces of advice (e.g., regarding fat quality and viscous fibers) were not associated with effects in this setting. Such advice is well-known, and the included participants were volunteers willing to improve blood lipids and, thus, plausibly well-informed on the subject already. It may also be due to chance, as the 95% CIs for β-coefficients for most pieces of dietary advice did in fact include a relevant improvement. Alternatively, the absence of associations may relate to the food matrices of consumed fatty foods, or certain pieces of advice may be less effective in the real-world setting when foods are not provided within a study protocol, and new dietary habits need to be established in the home environment. Dietary changes may require more support from dieticians or other health care providers regarding some foods. In addition, the quality of evidence for the included advice was not assessed a priori. Subsequent analyses revealed that some of the suggested dietary changes may, in fact, be ineffective, even under ideal conditions, or at least that their evidence bases are weak, related to risk of bias in published RCTs, heterogeneity of results, indirectness, imprecision, or risk of publication bias [5]. Other biases may also inflate results, such as conflicts of interest in RCTs [35], as well as systematic reviews [36], or issues with corrupt or outright fabricated data [37]. 

We acknowledge several limitations in this study. The dietary advice diary has not (yet) been validated against food records or biomarkers, and there was no other dietary assessment before or during the study. Therefore, we essentially lacked information regarding background diets and the extent of the performed changes. The population was rural and of mostly Caucasian origin, and their dietary habits likely resemble those of the overall Swedish population [38], with a high intake of saturated fat, salt, and sugar, and a low intake of fiber, fruits, vegetables, and fish compared with national dietary guidelines [39]. Hence, the results may not apply to other populations. We had limited power to draw conclusions regarding the associations of effects on blood lipids with individual pieces of advice. Notably, the randomized design applied only to (here mostly absent) between-group effects, whereas all other associations should be interpreted cautiously for causal inference. For instance, individuals willing to try more unusual dietary advice, such as turmeric or plant sterols, may be more prone to making other or more pronounced lifestyle changes as well. We performed multiple secondary analyses, which induces the risk of type I errors. Although participants were instructed not to discuss study details outside their households, we cannot rule out cross-over between study arms, which would bias results towards the null. A potential seasonal effect could also introduce bias towards higher blood lipids overall at the end of the study, as participants were included from May to October. Lastly, the inclusion of advice to limit fast carbohydrates (including sugar) in the control group only may also have reduced between-group differences, as sugar has a small detrimental effect also on LDL cholesterol [5]. Strengths of this study include its preregistered protocol and statistical analysis plan, partially blinded design, equal-intensity control group, provision of updated and detailed dietary advice, and minimal loss to follow-up.

Future interventions should focus on advice with clearly established effects, as well as more distinctly highlighting which foods should be replaced. Food records could be included in the intervention in a simplified format or performed within a subsample of participants to provide information about dietary intakes without compromising the pragmatic design. The suggested short-term effects of turmeric and plant sterols warrant further research, as do the potential effects of replacing unfiltered with filtered coffee.

## 5. Conclusions

Written food-based advice combined with feedback was ineffective for improving LDL cholesterol compared with other, neutral advice. Further studies are needed to establish which selection of dietary advice can be most effective for improving blood lipids in real-world primary care settings.

## Figures and Tables

**Table 1:** Baseline characteristics.

	All (*n* = 113) ^a^	Intervention Group (*n* = 56)	Control Group (*n* = 57)	*p*
Age (years)	66.1 (57.5–71.9)	66.0 (52.1–73.4)	66.1 (61.0–71.6)	0.45
Sex: female, male, (*n*, %)	78 (69%), 35 (31%)	38 (68%), 18 (32%)	40 (70%), 17 (30%)	0.84
Diabetes, *n* (%)	7 (6%)	3 (5%)	4 (7%)	1.00
Impaired fasting glucose (*n*, %)	9 (8%)	4 (7%)	5 (9%)	1.00
Body weight (kg)	77.7 (69.1–87.2)	77.9 (68.0–85.2)	77.0 (69.8–88.0)	0.70
BMI (kg/m^2^)	27.6 ± 4.5	27.4 ± 4.6	27.7 ± 4.4	0.72
Waist circumference (cm)	93.8 ± 12.1	93.3 ± 12.4	94.4 ± 11.9	0.64
SBP (mmHg)	139.7 ± 21.1	137.4 ± 20.4	142.0 ± 21.7	0.25
DBP (mmHg)	80.9 ± 10.2	79.9 ± 9.8	81.9 ± 10.5	0.31
LDL cholesterol (mmol/L)	3.94 ± 0.94	4.09 ± 0.96	3.79 ± 0.91	0.09
Total cholesterol (mmol/L)	5.68 ± 0.98	5.78 ± 1.03	5.57 ± 0.91	0.25
HDL cholesterol (mmol/L)	1.58 ± 0.36	1.58 ± 0.35	1.58 ± 0.38	0.96
Apolipoprotein B (g/L)	1.01 ± 0.22	1.03 ± 0.21	0.99 ± 0.22	0.26
Apolipoprotein AI (g/L)	1.43 ± 0.19	1.43 ± 0.20	1.43 ± 0.18	0.87
Apolipoprotein B: AI ratio	0.72 ± 0.18	0.73 ± 0.17	0.70 ± 0.18	0.38
Triglycerides (mmol/L)	1.1 (0.8–1.5)	1.15 (0.8–1.5)	1.1 (0.7–1.5)	0.53
Glucose ^b^ (mmol/L)	5.5 (5.1–5.7)	5.3 (5.0–5.7)	5.4 (5.2–5.7)	0.55
HbA1c (mmol/mol)	38 (36–41)	38 (35–41)	38 (37–41)	0.58

Abbreviations: DBP, diastolic blood pressure; SBP, systolic blood pressure. ^a^ *n* = 112/56/56 for all variables except age, sex, LDL cholesterol, and glucose. ^b^ *n* = 111/55/56. Data are mean ± standard deviation or median (interquartile range).

**Table 2:** Changes during the intervention (mean ± SD).

	Intervention Group (*n* = 56)	Control Group (*n* = 56)	*p*	Intervention Group (*n* = 56)	Control Group (*n* = 56)	*p*
	3 Weeks			6 Months		
Body weight (kg)	−0.36 ± 0.67	−0.81 ± 1.08	0.007	0.04 ± 2.22	−0.84 ± 2.57	0.06
BMI (kg/m^2^)	−0.13 ± 0.23	−0.28 ± 0.38	0.01	0.02 ± 0.79	−0.29 ± 0.87	0.051
Waist circumference (cm)	−2.16 ± 3.58	−1.90 ± 3.08	0.65	−3.75 ± 5.06	−4.44 ± 4.19	0.44
SBP (mmHg)	−4.59 ± 12.0	−5.05 ± 12.7	0.71	−3.16 ± 13.7	−3.37 ± 14.5	0.58
DBP (mmHg)	−1.96 ± 6.92	−1.65 ± 6.74	0.47	−0.39 ± 7.87	−0.96 ± 7.1	0.99
LDL cholesterol (mmol/L)	−0.21 ± 0.38	−0.11 ± 0.34 ^a^	0.45	−0.046 ± 0.47	0.020 ± 0.41 ^a^	0.70
Total cholesterol (mmol/L)	−0.19 ± 0.46	−0.14 ± 0.44	0.87	0.034 ± 0.49	0.079 ± 0.47	0.89
HDL cholesterol (mmol/L)	−0.009 ± 0.13	−0.005 ± 0.13	0.87	−0.054 ± 0.15	−0.014 ± 0.12	0.10
Apolipoprotein B (g/L)	−0.040 ± 0.09	−0.024 ± 0.09	0.84	−0.019 ± 0.10	−0.019 ± 0.09	0.54
Apolipoprotein AI (g/L)	−0.003 ± 0.10	−0.022 ± 0.10	0.27	0.036 ± 0.12	0.035 ± 0.09	0.93
Apolipoprotein B:AI ratio	−0.028 ± 0.07	−0.005 ± 0.07	0.18	−0.031 ± 0.09	−0.032 ± 0.06	0.43
Triglycerides (mmol/L)	0.002 ± 0.41	−0.030 ± 0.29	0.59	0.052 ± 0.43	0.044 ± 0.40	0.87
Glucose (mmol/L)	−0.007 ± 0.58	−0.12 ± 0.44	0.26	0.096 ± 0.75	−0.058 ± 0.47	0.17
HbA1c (mmol/mol)	−0.57 ± 1.49	−0.32 ± 1.39	0.28	−0.70 ± 1.81	−0.58 ± 1.79	0.80
**Change in Intervention Group Responders ^b^**						
	**3 Weeks**			**6 Months**		
LDL cholesterol (mmol/L)	−0.67 ± 0.27		5 × 10^−7 c^	−0.31 ± 0.38		0.01 ^c^

Abbreviations: DBP, diastolic blood pressure; SBP, systolic blood pressure. *p*-values are from general linear models and are adjusted for baseline values, BMI, age, and sex. ^a^ *n* = 57; ^b^ *n* = 14 in the intervention group with ≥10% in LDL cholesterol; ^c^ *p*-values are from *t*-tests compared with baseline means.

**Table 3:** Linear regressions of self-reported compliance to specific dietary advice ^a^ and effects on LDL cholesterol or triglycerides.

Intervention Group—LDL Cholesterol (*n* = 56)					
		3 Weeks		6 Months	
Dietary Advice	Adherence (Maximum 21 Days per Advice), Median (IQR)	Beta (95% CI) in mmol/L per Daily Dietary Change	*p*	Beta (95% CI) in mmol/L per Daily Dietary Change	*p*
Total no. of reported daily dietary changes (out of maximum 273)	56 (32.75–92)	−0.002 (−0.004, 0.000)	0.051	−0.002 (−0.004, 0.001)	0.26
Oils (replacing solid fats)	0.5 (0–7.5)	−0.003 (−0.019, 0.012)	0.69	0.000 (−0.019, 0.019)	0.98
Nuts and seeds	4 (0–11)	0.002 (−0.012, 0.017)	0.73	−0.004 (−0.022, 0.013)	0.61
Low−fat dairy products	0.5 (0–15)	−0.008 (−0.021, 0.006)	0.25	−0.016 (−0.032, −0.000)	0.048
Foods with added plant sterols	0 (0–0)	−0.027 (−0.047, −0.007)	0.009	−0.016 (−0.042, 0.010)	0.22
Pulses/soy (replacing meat and eggs)	0 (0–3.75)	−0.014 (−0.043, 0.015)	0.32	−0.007 (−0.042, 0.029)	0.71
Probiotics, e.g., A-fil ^b^ and yogurt	5.5 (0–15)	−0.006 (−0.019, 0.008)	0.41	−0.001 (−0.018, 0.016)	0.92
Oats, barley, and rye	3 (0–15.75)	−0.006 (−0.020, 0.007)	0.32	0.004 (−0.012, 0.021)	0.58
Fruits, berries, and vegetables (including avocado and tomato)	5.5 (0–14.75)	−0.002 (−0.015, 0.012)	0.79	0.001 (−0.016, 0.017)	0.91
Filtered coffee (replacing unfiltered)	0 (0–6.5)	−0.012 (−0.025, 0.001)	0.08	−0.015 (−0.031, 0.000)	0.06
Tea (preferably green)	3 (0–10.75)	−0.011 (−0.026, 0.004)	0.13	0.001 (−0.018, 0.019)	0.95
Turmeric	0.5 (0–9.5)	−0.019 (−0.034, −0.005)	0.01	−0.012 (−0.030, 0.007)	0.21
Garlic	0.5 (0–5)	−0.005 (−0.027, 0.017)	0.68	−0.012 (−0.039, 0.015)	0.36
Dark chocolate	1.5 (0–6.5)	−0.009 (−0.028, 0.010)	0.36	−0.006 (−0.029, 0.018)	0.64
**Control Group—Triglycerides (*n* = 57)**					
		**3 Weeks**		**6 Months**	
**Dietary Advice**	**Adherence (Maximum 21 Days per Advice), Median (IQR)**	**Beta (95% CI) in mmol/L per Daily** **Dietary Change**	***p***	**Beta (95% CI) in mmol/L per Daily** **Dietary Change**	***p***
Total no. of reported daily dietary changes (out of maximum 63)	14 (7–25)	−0.005 (−0.012, 0.002)	0.15	−0.003 (−0.013, 0.006)	0.48
Less alcohol	0 (0–2)	−0.007 (−0.031, 0.016)	0.53	−0.013 (−0.046, 0.019)	0.42
Less carbohydrates	9 (3–14.5)	−0.014 (−0.024, −0.003)	0.01	−0.008 (−0.023, 0.008)	0.33
Fatty fish	4 (0–8)	0.004 (−0.012, 0.020)	0.59	0.003 (−0.019, 0.025)	0.76

Abbreviations: CI, confidence interval; IQR, interquartile range. *p*-values are from unadjusted linear regressions. ^a^ Daily reported changes from habitual dietary habits. ^b^ A-fil is a Swedish soured milk containing *L. acidophilus* and other species of bacteria.

## Data Availability

The full dataset will not be made available because ethical permissions do not allow it, but fully anonymized data and the analytic code may be shared upon reasonable request.

## Supplementary Materials

The following supporting information can be downloaded at: https://www.mdpi.com/article/10.3390/nu14051022/s1, Figure S1: Dietary advice given in the intervention (LDL cholesterol) and control (triglycerides) groups; Table S1: Standardized letters with feedback.
