# Supplementary material for: Effectiveness of Written Dietary Advice for Improving Blood Lipids in Primary Care Adults—A Pragmatic Randomized Controlled Trial (MYDICLIN)
Source: Nutrients. 2022 Feb 28;14(5):1022. doi: 10.3390/nu14051022 (PMC8912386; doi:10.3390/nu14051022)
Supplement: Supplementary file 1 [file nutrients-14-01022-s001.zip › nutrients-1546326-supplementary.pdf]

nutrients

MDPI

Online Supplementary Material – Rydell et al.

Effectiveness of written dietary advice for improving blood lipids in primary care adults – a pragmatic randomized controlled trial (MYDICLIN)

Supplemental Table S1. Standardized letters with feedback.

**3 weeks**<br><br>**Intervention group, clear effect (≥ 10% reduction in LDL cholesterol)**
Dear participant in the MYDICLIN study,<br><br>Congratulations! You have managed to lower your blood lipids considerably. Well done! You have succeeded in reducing your risk of future cardiovascular disease. Continue with the changes you have made, if you think they work well for you. Maybe there is something further you can improve up until the follow-up at 6 months?<br><br>Attached, you can see how your blood lipids have improved since the study start and how big a percentage change in LDL cholesterol this equals. Please write down the values in your study form.
**Control group, clear effect (≥ 10% reduction in triglycerides)**
Dear participant in the MYDICLIN study,<br><br>Congratulations! You have managed to lower your blood lipids considerably. Well done! You have succeeded in reducing your risk of future cardiovascular disease.<br><br>Continue with the changes you have made, if you think they work well for you. Maybe there is something further you can improve up until the follow-up at 6 months?

Attached, you can see how your blood lipids have improved since the study start and how big a percentage change in triglycerides this equals. Please write down the values in your study form.
**Intervention group, modest effect (> 0 to < 10% reduction in LDL cholesterol)**
Dear participant in the MYDICLIN study,<br><br>You have managed to lower your blood lipids to some extent. Well done! Please continue with the changes you have made. Even small improvements in blood lipids can reduce your risk of future cardiovascular disease.<br><br>That the effect has not been larger for you may depend on genetic factors, certain random variation between samples, or that the changes you have made are not so considerable compared with your previous diet. Some dietary changes may also take longer than three weeks to reach full effect on blood lipids. Maybe there is something further you can improve up until the follow-up visit at 6 months?<br><br>Attached, you can see how your blood lipids have improved since the study start and how big a percentage change in LDL cholesterol this equals. Please write down the values in your study form.
**Control group, modest effect (> 0 to < 10% reduction in triglycerides)**
Dear participant in the MYDICLIN study,<br><br>You have managed to lower your blood lipids to some extent. Well done! Please continue with the changes you have made. Even small improvements in blood lipids can reduce your risk of future cardiovascular disease.<br><br>That the effect has not been larger for you may depend on genetic factors, certain random variation between samples, or that the changes you have made are not so considerable compared with your previous diet, or maybe your levels were so low before the study, that little further improvement was

possible. Some dietary changes may also take longer than three weeks to reach full effect on blood lipids. Maybe there is something further you can improve up until the follow-up visit at 6 months?<br><br>Attached, you can see how your blood lipids have improved since the study start and how big a percentage change in triglycerides this equals. Please write down the values in your study form.
**Intervention group, no effect (≤ 0% reduction in LDL cholesterol)**
Dear participant in the MYDICLIN study,<br><br>Unfortunately, you have not managed to lower your blood lipids.<br><br>That there was no improvement may depend on genetic factors, or that the changes you have made are not so considerable compared with your previous diet, or on certain random variation between samples, which could obscure a true improvement. Some dietary changes may also take longer than three weeks to reach full effect on blood lipids. Maybe these particular dietary changes didn't suit you, regarding effects on blood lipids.<br><br>Maybe you can to try to make more or larger changes, or try if another dietary change suits you better, in order to try to achieve an improvement before the follow-up? If your blood lipids have clearly deteriorated, it may be better to return to your usual diet as it was before the study start.<br><br>Attached, you can see how your blood lipids have changed (or not) since the study start and how big a percentage change in LDL cholesterol this equals. Please write down the values in your study form.
**Control group, no effect (≤ 0% reduction in triglycerides)**
Dear participant in the MYDICLIN study,<br><br>Unfortunately, you have not managed to lower your blood lipids.<br><br>That there was no improvement may depend on genetic factors, or that the changes you have made are not so considerable compared with your previous diet, or on certain random variation between

samples, which could obscure a true improvement. Some dietary changes may also take longer than three weeks to reach full effect on blood lipids. Maybe these particular dietary changes didn't suit you, regarding effects on blood lipids, or maybe your levels were so low before the study, that little further improvement was possible.<br><br>Maybe you can to try to make more or larger changes, or try if another dietary change suits you better, in order to try to achieve an improvement before the follow-up? If your blood lipids have clearly deteriorated, it may be better to return to your usual diet as it was before the study start.<br><br>Attached, you can see how your blood lipids have changed (or not) since the study start and how big a percentage change in triglycerides this equals. Please write down the values in your study form.
**6 months**<br><br>**Intervention group, clear effect (≥ 10% reduction in LDL cholesterol)**
Dear participant in the MYDICLIN study,<br><br>Congratulations! You have, after six months, managed to lower your blood lipids considerably. Well done! You have succeeded in reducing your risk of future cardiovascular disease.<br><br>Attached, you can see how your blood lipids have improved since the study start and how big a percentage change in LDL cholesterol this equals. Please write down the values in your study form.<br><br>Attached is also the dietary advice given to the other study group. That advice does not affect LDL cholesterol much, but instead focuses on lowering triglycerides. That's another lipoprotein sampled from fasting blood samples. In existing guidelines, most focus is on LDL cholesterol, but triglycerides may also affect the risk of future cardiovascular disease, and dietary advice differs depending on which risk factor you want to target.

Attached is also a small survey in which you may write comments regarding the study, or state if you have questions and wish to be contacted.
**Control group, clear effect (≥ 10% reduction in triglycerides)**
Dear participant in the MYDICLIN study,<br><br>Congratulations! You have, after six months, managed to lower your blood lipids considerably. Well done! You have succeeded in reducing your risk of future cardiovascular disease.<br><br>Attached, you can see how your blood lipids have improved since the study start and how big a percentage change in triglycerides this equals. Please write down the values in your study form.<br><br>Attached is also the dietary advice given to the other study group. That advice does not affect triglycerides much, but instead focuses on lowering LDL cholesterol. That's another lipoprotein which affects the risk of cardiovascular disease. In existing guidelines, most focus is on LDL cholesterol, but triglycerides may also affect the risk of future cardiovascular disease, and dietary advice differs depending on which risk factor you want to target.<br><br>Attached is also a small survey in which you may write comments regarding the study, or state if you have questions and wish to be contacted.
**Intervention group, modest effect (> 0 to < 10% reduction in LDL cholesterol)**
Dear participant in the MYDICLIN study,<br><br>You have, after six months, managed to lower your blood lipids to some extent. Well done! Even a small improvement in blood lipids can reduce your risk of future cardiovascular disease.<br><br>Attached, you can see how your blood lipids have improved since the study start and how big a percentage change in LDL cholesterol this equals. Please write down the values in your study form.

Attached is also the dietary advice given to the other study group. That advice does not affect LDL cholesterol much, but instead focuses on lowering triglycerides. That's another lipoprotein sampled from fasting blood samples. In existing guidelines, most focus is on LDL cholesterol, but triglycerides may also affect the risk of future cardiovascular disease, and dietary advice differs depending on which risk factor you want to target.<br><br>Attached is also a small survey in which you may write comments regarding the study, or state if you have questions and wish to be contacted.
**Control group, modest effect (> 0 to < 10% reduction in triglycerides)**
Dear participant in the MYDICLIN study,<br><br>You have, after six months, managed to lower your blood lipids to some extent. Well done! Even a small improvement in blood lipids can reduce your risk of future cardiovascular disease.<br><br>Attached, you can see how your blood lipids have improved since the study start and how big a percentage change in triglycerides this equals. Please write down the values in your study form.<br><br>Attached is also the dietary advice given to the other study group. That advice does not affect triglycerides much, but instead focuses on lowering LDL cholesterol. That's another lipoprotein which affects the risk of cardiovascular disease. In existing guidelines, most focus is on LDL cholesterol, but triglycerides may also affect the risk of future cardiovascular disease, and dietary advice differs depending on which risk factor you want to target.<br><br>Attached is also a small survey in which you may write comments regarding the study, or state if you have questions and wish to be contacted.
**Intervention group, no effect (≤ 0% reduction in LDL cholesterol)**
Dear participant in the MYDICLIN study,

Unfortunately, you have not managed to lower your blood lipids after six months.<br><br>Attached, you can see how your blood lipids have changed (or not) since the study start and how big a percentage change in LDL cholesterol this equals. Please write down the values in your study form.<br><br>Attached is also the dietary advice given to the other study group. That advice does not affect LDL cholesterol much, but instead focuses on lowering triglycerides. That's another lipoprotein sampled from fasting blood samples. In existing guidelines, most focus is on LDL cholesterol, but triglycerides may also affect the risk of future cardiovascular disease, and dietary advice differs depending on which risk factor you want to target.<br><br>Attached is also a small survey in which you may write comments regarding the study, or state if you have questions and wish to be contacted.
**Control group, no effect (≤ 0% reduction in triglycerides)**
Dear participant in the MYDICLIN study,<br><br>Unfortunately, you have not managed to lower your blood lipids after six months.<br><br>Attached, you can see how your blood lipids have changed (or not) since the study start and how big a percentage change in triglycerides this equals. Please write down the values in your study form.<br><br>Attached is also the dietary advice given to the other study group. That advice does not affect triglycerides much, but instead focuses on lowering LDL cholesterol. That's another lipoprotein which affects the risk of cardiovascular disease. In existing guidelines, most focus is on LDL cholesterol, but triglycerides may also affect the risk of future cardiovascular disease, and dietary advice differs depending on which risk factor you want to target.<br><br>Attached is also a small survey in which you may write comments regarding the study, or state if you have questions and wish to be contacted.

Supplemental Figure S1. Dietary advice given in the A. intervention (LDL cholesterol) and B. control (triglycerides) groups.

Dietary advice to lower blood lipids

Great that you want to improve your dietary habits! Choose which dietary changes you want to make. **Make an X on the back of this paper** for each day that you make a change compared with your usual dietary habits. If you, e.g., start eating oatmeal, make an X in the row for "Oats, barley and rye", one X for each relevant day, but not if you regularly eat oatmeal already. The results are individual and may depend on genetic factors and your previous diet. If you perform several changes simultaneously, your LDL cholesterol may be reduced (in the best case) by up to 30%. A smaller reduction may also improve your risk of future cardiovascular disease. You don't need to eat excessive amounts of any single food and you don't need to lose weight in order to have an effect.

The following foods may lower cholesterol levels, by reduced production in the liver ( ), reduced intestinal uptake ( ), increased uptake from blood ( ) or unclear mechanisms (•):

- Fatty foods high in unsaturated fatty acids, e.g., rapeseed oil, olive oil, nuts, seeds, and avocado can clearly lower LDL cholesterol, especially when replacing foods high in saturated fatty acids, e.g., butter, coconut, palm oil, or so called *trans* fats ("partially hydrogenated vegetable fat", which can be part of bakeries and other fatty foods).
- Replacing high-fat foods, e.g., dairy, with lower-fat varieties produces a somewhat smaller effect.
- Foods with added plant sterols (some spreads and drinks) can lower LDL cholesterol. The effect is clear from at least two daily servings. Lowering cholesterol intake (eggs and animal foods) can also reduce LDL cholesterol, but the effect is somewhat smaller.
- Probiotics, with healthy bacteria, such as A-fil and yogurt, can lower LDL cholesterol moderately.
- Food containing fiber (especially so called viscous fiber) such as oats, barley, rye, and whole-grain products, pulses (beans, lentils, chickpeas, and peas), fruits (e.g., apples, pears, bananas, and citrus fruits) and vegetables (e.g., broccoli and cabbage), nuts and seeds have some effect.
- Berries (especially blue, red, or violet), which contain so called anthocyanins, have some effect.
- Tomatoes contain among other things lycopene and seem to lower LDL cholesterol somewhat.
- Vegetable protein (e.g., soy products) can possibly lower LDL cholesterol somewhat.
- Replacing unfiltrered (e.g., boiled) coffee with filtrered (e.g., brewed) can lower LDL cholesterol clearly, as the cholesterol-raising substances kahweol and cafestol remain in the coffee grounds.
- Tea (especially green) contain polyphenols and may possibly lower LDL cholesterol somewhat.
- Turmeric contains the yellow substance curcumin and may possibly lower LDL cholesterol moderately.
- Garlic contains allicin and may lower total and perhaps also LDL cholesterol somewhat.
- Cocoa contains polyphenols and may possibly lower LDL cholesterol somewhat, but this probably only applies to certain dark chocolates, as sugar may have the opposite effect.

Which pieces of advice suit you? How low can you go?

(**A**)

Dietary advice to lower blood lipids

Great that you want to improve your dietary habits! Choose which dietary changes you want to make. **Make an X on the back of this paper** for each day that you make a change compared with your usual dietary habits. If you, e.g., start eating fatty fish, you mark an X in the row for "Fatty fish", one X for each relevant day, but not if you regularly eat fatty fish already. If you perform several changes simultaneously, your triglycerides may be reduced by 30% or more, depending on your previous levels and diet. You don't need to eat excessive amounts of any single food and you don't need to lose weight in order to have an effect.

The following dietary changes can lower triglycerides, by reduced production of so called VLDL particles in the liver:

- To reduce alcohol intake.
- To reduce the amount of carbohydrate, especially fast carbohydrates (e.g., sugar, refined grains, white rice, potatoes, and pasta).
- To eat more fatty fish, which contains omega-3 fatty acids.

Which pieces of advice suit you? How low can you go?

(B)
